# TRPV1 alleviates osteoarthritis by inhibiting M1 macrophage polarization via Ca^2+^/CaMKII/Nrf2 signaling pathway

**DOI:** 10.1038/s41419-021-03792-8

**Published:** 2021-05-18

**Authors:** Zhongyang Lv, Xingquan Xu, Ziying Sun, Yannick Xiaofan Yang, Hu Guo, Jiawei Li, Kuoyang Sun, Rui Wu, Jia Xu, Qing Jiang, Shiro Ikegawa, Dongquan Shi

**Affiliations:** 1 State Key Laboratory of Pharmaceutical Biotechnology, Department of Sports Medicine and Adult Reconstructive Surgery, Nanjing Drum Tower Hospital, The Affiliated Hospital of Nanjing University Medical School, 321 Zhongshan Road, Nanjing, 210008 Jiangsu People’s Republic of China; 2 Laboratory for Bone and Joint Disease, Model Animal Research Center (MARC), Nanjing University, Nanjing, 210093 Jiangsu People’s Republic of China; 3 Drum Tower of Clinical Medicine, Nanjing Medical University, Nanjing, 210008 Jiangsu People’s Republic of China; 4 Laboratory for Bone and Joint Diseases, RIKEN Center for Integrative Medical Science (IMS, RIKEN), Tokyo, 108-8639 Japan

**Keywords:** Inflammation, Osteoarthritis

## Abstract

Osteoarthritis (OA) is the major course of joint deterioration, in which M1 macrophage-driven synovitis exacerbates the pathological process. However, precise therapies for M1 macrophage to decrease synovitis and attenuate OA progression have been scarcely proposed. Transient receptor potential vanilloid 1 (TRPV1) is a cation channel that has been implicated in pain perception and inflammation. In this study, we investigated the role of TRPV1 in the M1 macrophage polarization and pathogenesis of OA. We demonstrated that TRPV1 expression and M1 macrophage infiltration were simultaneously increased in both human and rat OA synovium. More than 90% of the infiltrated M1 macrophages expressed TRPV1. In the rat OA model, intra-articular injection of capsaicin (CPS), a specific TRPV1 agonist, significantly attenuated OA phenotypes, including joint swelling, synovitis, cartilage damage, and osteophyte formation. CPS treatment markedly reduced M1 macrophage infiltration in the synovium. Further mechanistic analyses showed that TRPV1-evoked Ca^2+^ influx promoted the phosphorylation of calcium/calmodulin-dependent protein kinase II (CaMKII) and facilitated the nuclear localization of nuclear factor-erythroid 2-related factor 2 (Nrf2), which ultimately resulted in the inhibition of M1 macrophage polarization. Taken together, our findings establish that TRPV1 attenuates the progression of OA by inhibiting M1 macrophage polarization in synovium via the Ca^2+^/CaMKII/Nrf2 signaling pathway. These results highlight the effect of targeting TRPV1 for the development of a promising therapeutic strategy for OA.

## Introduction

Osteoarthritis (OA) is the most common form of joint deterioration, manifesting several symptoms that weaken the quality of life, such as pain, joint stiffness, and dysfunction^1^. OA increases socioeconomic burden due to growing prevalence and incidence^2^. However, there is no fundamental treatment of OA as its pathogenesis is not sufficiently clarified.

Synovial inflammation (synovitis) is highly correlated with the progression and severity of OA^3,4^. Recently, OA is well established as a low-grade inflammatory disease primarily mediated by the activity of innate immune cells, especially macrophages^5^. Macrophages can be generally classified as inflammatory M1 macrophages and anti-inflammatory M2 macrophages^6^. Emerging evidence reveals that the M1 macrophage polarization in the synovium plays a critical role in the development of OA^7,8^. However, conditional depletion of macrophages failed to mitigate OA and instead resulted in increased inflammatory cytokines in joint synovial fluid^9^, highlighting the difficulty of targeting the highly complex immune microenvironment in OA. Therefore, a targeted approach to inhibit M1 macrophage polarization and alleviate OA is awaiting to be developed.

Transient receptor potential vanilloid 1 (TRPV1) is a nonspecific cation channel that can be activated by various thermal, mechanical, and chemical stimuli^10^. The role of TRPV1 in pain perception has been investigated in a variety of diseases^11–13^. Intra-articular injection of a TRPV1 agonist significantly suppresses pain in an OA model and OA patients^14,15^. Furthermore, TRPV1 activation is also involved in regulating immune cell activity, including the activation of CD4-positive T cells and the proinflammatory phenotype polarization of macrophages (M1)^16,17^. In contrast, a recent study has shown that TRPV1 modulates M1/M2 macrophage polarization and decreases the levels of M1 macrophage markers, inducible nitric oxide synthase (iNOS) and interleukin-6 (IL-6), to promote dopaminergic neurons survival in a Parkinson’s disease model^18^. This apparent divergent role of TRPV1 on M1 macrophage polarization remains to be explored. Interestingly, the immunoreactivity of TRPV1 is increased in the OA synovium, with its majority localized to CD68-positive macrophages^19^. However, whether TRPV1 can regulate M1 macrophage polarization and the role of TRPV1 in OA synovitis remain unknown.

In this study, we examined the human and rat OA synovium and observed a significant increase of TRPV1 expression and M1 macrophage infiltration. In a rat OA model of the radial transection of the medial meniscus, intra-articular injection of capsaicin (CPS), a specific TRPV1 agonist, significantly decreased M1 macrophage polarization in synovium and reduced synovitis, and sequentially attenuated cartilage deterioration and osteophyte formation. Further, TRPV1 inhibited M1 macrophage polarization via the Ca^2+^/calcium/calmodulin-dependent protein kinase II/nuclear factor-erythroid 2-related factor 2 (Ca^2+^/CaMKII/Nrf2) signaling pathway. These findings suggest that TRPV1 is a novel therapeutic target reducing synovitis and alleviating OA.

## Materials and methods

### Clinical specimen

The study protocol was approved by the Ethical Committee of the Nanjing Drum Tower Hospital, the Affiliated Hospital of Nanjing University Medical School (2009022). Normal synovial tissues were obtained from three patients (56–71 years old) without OA history who underwent lower limb amputation due to a traffic accident. OA synovial tissues were obtained from age-matched six patients (59–77 years old, Kellgren-Lawrence grade 4) who suffered from OA and underwent total knee arthroplasty. All of the patients accepted informed consent before their enrollment.

### Animals

All animal experiments were authorized and performed in accordance with the Animal Care and Use Committee of Nanjing Drum Tower Hospital, The Affiliated Hospital of Nanjing University (2019AE02010). We have complied with all relevant codes of ethics.

Adult male Sprague–Dawley (SD) rats (2 months, *n* = 36) were obtained from the Animal Center of the Nanjing Medical University (Jiangsu, China). The number of rats in each group was chosen based on the calculation formula reported by Charan and Kantharia^20^. After a 1-week acclimation period, 24 rats underwent surgery for the radial transection of the medial meniscus to generate an OA model^21^, while the remaining 12 rats received a sham operation. Then, the rats were randomly grouped into either the 4 or 8 weeks of the sham, OA, or OA with CPS treatment (OA + CPS) groups (*n* = 6, each group). Intra-articular injection of 50 μL of 50 μM CPS (#ab141000, Abcam, Cambridge, USA) was administered twice a week for 4 or 8 weeks in the OA + CPS group, while rats in the OA group were injected with 50 μL phosphate-buffered saline (PBS). During this procedure, the knee diameter of the rats in each group was monitored by an electronic vernier caliper.

### Cell culture

RAW264.7 cells were purchased from the Cell Bank of Type Culture Collection of Chinese Academy of Science (Shanghai, China) and cultured in Dulbecco’s modified Eagle’s medium (Gibco, Carlsbad, CA) supplemented with 10% fetal bovine serum (Gibco) and 1% penicillin and streptomycin (Gibco) at 37 °C and 5% CO_2_ condition. The cells were induced for the M1 macrophage polarization with 50 ng/mL lipopolysaccharide (LPS) (#L2630, Sigma-Aldrich, St. Louis, MO, USA). To assess the role of TRPV1 in M1 macrophage polarization, 50 μM CPS was used to activate TRPV1. To study the regulation of the CaMKII phosphorylation, cells were induced by LPS with or without CPS for 5, 15, and 30 min. To evaluate the Nrf2 nuclear translocation, the cells were pretreated with 500 μM ethylenediaminetetraacetic acid (EDTA) (#1340, Biofroxx, Germany), 20 μM KN-93 (#HY-15465, MCE-MedChemExpress, New Jersey, USA), or 5 μM ML385 (#HY-100523, MCE) for 1 h, followed by the treatment of LPS and CPS for 3 h. To evaluate the expression of M1 macrophage markers, the cells were treated with LPS with or without CPS for 24 and 48 h and the messenger RNA (mRNA), and protein expression levels of M1 macrophage markers were analyzed.

### Histological analysis

After rats were sacrificed, their affected knee joints were fixed in 4% paraformaldehyde (PFA) for 3 days, and the samples then underwent decalcification in 10% EDTA (#1340, Biofroxx) solution for 2 months. The decalcified tissues were then embedded in paraffin blocks and cut into 5-μm coronal slides by a microtome (Thermo, Germany). Tissue sections from each experimental group were stained by hematoxylin and eosin (H&E) (#C0105S Beyotime, Shanghai, China) and safranin O/fast green (#G1371, Solarbio, Beijing, China). The Osteoarthritis Research Society International (OARSI) scoring and synovitis scoring^22^ were assessed by two individuals who were ignorant of the animals’ treatment. The M1 macrophage polarization was assessed by immunofluorescence staining of the synovium.

### Immunofluorescence staining

Histological slides of rat knee tissues were deparaffinized in xylene and graded into water using different concentrations of ethanol ranging from 100 to 50%. The slides were blocked with 5% bovine serum albumin (BSA) for 1 h at room temperature and incubated with primary antibodies (1:200, dilution) for CD14 (#17000-1-AP, Proteintech, Wuhan, China), CD80 (#66406-1-Ig, Proteintech), CD163 (#16646-1-AP, Proteintech), CD206 (#18704-1-AP, Proteintech), F4/80 (#27044-1-AP, Proteintech), iNOS (#13120S, Cell Signaling Technology, USA), and TRPV1 (#66983-1-Ig, Proteintech) overnight at 4 °C. For co-immunostaining, two primary antibodies from distinct species were co-incubated on slides. The slides were washed with PBS and incubated with fluorescein isothiocyanate (FITC)- or tetramethylrhodamine-5-(and 6)-isothiocyanate-conjugated secondary antibody for 1 h at room temperature. The nuclei were labeled by incubation with 4′,6-diamidino-2-phenylindole, and fluorescence images of randomly selected fields were obtained with a fluorescence microscope (Zeiss, Germany). In the cytological immunofluorescence, the cells were fixed in 4% PFA and permeated by 0.1% Triton X-100 for 15 min. After blocking with 5% BSA, the slides were incubated with primary antibody for Nrf2 (#16396-1-AP, 1:200, Proteintech). The remaining steps were the same as those used for the histological immunofluorescence staining.

### Immunohistochemical staining

Following paraffin dehydration and clearing and antigen retrieval, endogenous peroxidase activity was quenched via incubation with 3% (v/v) H_2_O_2_ for 15 min. Primary antibodies (1:200, dilution) for iNOS (#13120 S, Cell Signaling Technology) and TRPV1 (#22686-1-AP, Proteintech) were used, followed by incubation with a horseradish peroxidase-conjugated goat anti-rabbit immunoglobulin G (IgG) (1:1000, #BL003A, Biosharp, China) secondary antibody. Immunohistochemical staining was visualized using an ultra-sensitive DAB Kit (#1205250, Typing, China). Nonimmune IgG was used instead of the primary antibody as a negative control.

### Micro-computed tomography (micro-CT)

After fixed in 4% PFA, the microstructure of rat knee joints was analyzed using a micro-CT scanner (mCT80; Scanco Medical AG) with 80-μm voxel resolution. The reconstruction images were acquired by Scanco Medical software.

### Measurement of the intracellular Ca^2+^ concentration

Intracellular Ca^2+^ concentration was measured using a Ca^2+^ probe, Fluo-4 AM (#S1060, Beyotime) as previously described^23,24^. RAW264.7 cells were grown on a 12-well plate and serum-starved for 12 h before the incubation of Fluo-4 AM (2.5 μM) in a serum-free medium for 30 min. Under the Ca^2+^-free medium condition, the Fluo-4 AM-loaded RAW264.7 cells were treated with CaCl_2_ (2 mM), CPS (50 μM), or CaCl_2_ (2 mM) + CPS (50 μM) for 10, 30, 60, and 90 min. The fluorescence images were obtained with a fluorescence microscope (Zeiss, Germany).

### Enzyme-linked immunosorbent assay (ELISA)

The IL-6 levels in the supernatant of cultured RAW264.7 cells were detected using an IL-6 ELISA Kit according to the manufacturer’s protocol (#PI326, Beyotime Biotechnology, China). The absorbance at 450 nm was measured by a microplate reader. The concentrations of IL-6 in the supernatant were normalized to the cell count.

### Western blot

The total protein was extracted from RAW264.7 cells using RIPA (#R0010, Solarbio) lysis buffer containing 1 mM phenylmethanesulfonyl fluoride (#329-98-6, Solarbio) and 1 mM phosphatase inhibitor cocktail (#B15002, Bimake, USA). Cytosolic and nuclear proteins were purified from the induced cells separately, using a Nuclear and Cytosolic Extraction Reagent Kit (#R0050, Solarbio). Lysate protein concentrations were determined by BCA Protein Assay Kit (#23225, Thermo Scientific, MA, USA). Proteins were separated on 10% (w/v) sodium dodecyl sulfate-polyacrylamide gel (#PG112, EpiZyme, Shanghai, China) electrophoresis and were subsequently transferred onto polyvinylidene fluoride membranes (#IPVH00010, Millipore, USA) according to the standard procedures. After the membranes were blocked with 5% (w/v) milk (#1172GR500, Biofroxx) for 1 h at room temperature, the membranes were incubated in primary antibodies (1:1000 dilution) for Nrf2 (#16396-1-AP, Proteintech), glyceraldehyde 3-phosphate dehydrogenase (#5174S, Cell Signaling Technology), histone H3 (#4499S, Cell Signaling Technology), p-CaMKII (#12716S, Cell Signaling Technology), CaMKII (#12666-2-AP, Proteintech), iNOS (#13120S, Cell Signaling Technology), cyclooxygenase 2 (COX2) (#12282S, Cell Signaling Technology), and β-actin (#4970S, Cell Signaling Technology). A horseradish peroxidase-conjugated goat anti-rabbit/mouse IgG (1:5000, #BL003A or #BL001A, Biosharp) was used as a secondary antibody. All signals were detected by the ChemiDocXRS + Imaging System (Tanon, Shanghai, China). Quantitative analysis of protein densitometry was performed using Image J (version 1.8.0).

### Quantitative real-time polymerase chain reaction (qPCR)

Total RNAs were isolated using the RNA-quick Purification Kit (#RN001, ES Science, Shanghai, China) according to the manufacturer’s instructions. qPCR was conducted by magnifying 20 μL of diluted complementary DNA with the SYBR Green Q-PCR Kit (#Q411-02, Vazyme, Nanjing, China) on a LightCycler 480 PCR System (Roche, Switzerland). The primer sequences of mRNAs used are shown in Supplementary Table 1.

### Flow cytometry

After LPS treatment with or without CPS for 48 h, RAW264.7 cells were suspended in PBS and then stained with a mixture of fluorochrome-conjugated anti-mouse antibodies: F4/80-FITC (#123107, BioLegend, USA) and CD80-APC (#104713, BioLegend). After the cells were washed three times, they were diluted in flow buffer and run through a BD AccuriC6 Plus Flow CytoMeter (BD Biosciences, USA). Data were analyzed with the FlowJo software (version 10).

### Alamar Blue assay and Cell Counting Kit-8 (CCK8) assay

The proliferation capacity of RAW264.7 cells was assessed by Alamar Blue (#A7631, Solarbio) and CCK8 (#CK04, Dojindo, Shanghai, China) assay according to the manufacturer’s instructions after LPS treatment with or without CPS for 24 h. For Alamar Blue assay, the fluorescence at 545 nm upon excitation with radiation at 590 nm was measured. For CCK8 assay, the absorbance at 450 nm wavelength was detected.

### 5-Ethynyl-2′-deoxyuridine (EdU) assay

The EdU Reagent Kit (#C10310-1, Ribobio, Guangzhou, China) was used to evaluate cell proliferation. Briefly, RAW264.7 cells were treated with LPS with or without CPS for 24 h, followed by 10 μM EdU incubation for 2 h. The following steps were carried out according to the manufacturer’s protocol, and images were obtained with a fluorescence microscope (Zeiss).

### Scratch assay and Transwell migration assay

The effect of CPS on macrophage migration was assessed by in vitro Scratch assay and Transwell migration assay as previously reported^25^. Briefly, scratched wound lines were created by a 200 μL micropipette tip when the confluence of RAW264.7 cells reached 80%. Then, cells were treated by LPS with or without CPS for 48 h. For Transwell migration assay, RAW264.7 cells were seeded in the Transwell cell culture chambers with 8 μm pores, in the presence or absence of LPS or CPS. After incubation for 24 h, migrated cells were stained with crystal violet for the obtain of images.

### Statistical analyses

The SPSS software (version 25.0) and GraphPad Prism software (version 8.0) were used for the statistical analysis. Quantitative data are representative of at least three independent experiments. No samples or animals were excluded from the analysis. Shapiro–Wilk method was used to estimate the normal distribution of data and Levene method was used to test the homogeneity of variance. Unpaired two-tailed Student’s *t* test was used to compare mean values between two groups. For comparisons more than two groups, one-way analysis of variance, followed by Tukey’s multiple comparison tests were used. The data were presented as mean values ± SD. *P* < 0.05 was considered statistically significant.

## Results

### TRPV1 is highly expressed in M1 macrophage in the OA synovium

**Fig. 1:**
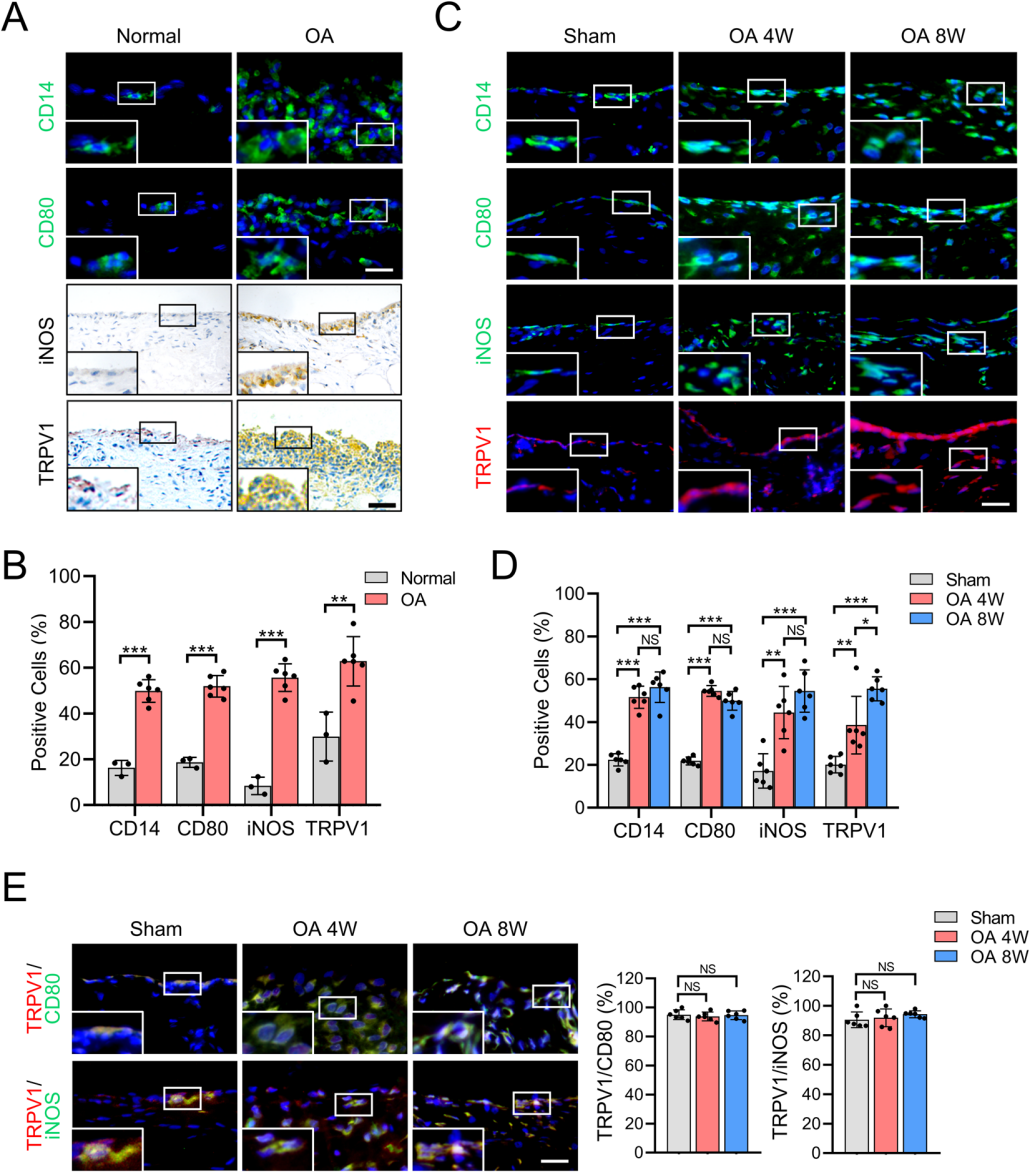
TRPV1 expression and M1 macrophage infiltration in the synovium of OA patients and rat OA model. **A** Immunofluorescence images for CD14 and CD80 staining, and immunohistochemical staining for iNOS and TRPV1 in normal and OA human synovium. **B** Quantification of CD14-, CD80-, iNOS-, and TRPV1-positive cells as a proportion of total cells in normal (*n* = 3) and OA (*n* = 6) human synovium. **C** Immunofluorescence images for CD14, CD80, iNOS, and TRPV1 in the synovium of sham and rats at 4 and 8 weeks after the radial transection of the medial meniscus. **D** Quantification of CD14-, CD80-, iNOS-, and TRPV1-positive cells as a proportion of total cells in the synovium of sham and 4- and 8-week-old OA rats (*n* = 6). **E** Co-immunostaining of TRPV1 with CD80 or iNOS in the synovium of sham and 4- and 8-week-old OA rats, and quantitative analysis of TRPV1-positive macrophages compared to total CD80- or iNOS-positive cells in the synovium of sham and 4- and 8-week-old OA rats (*n* = 6). Enlarged image is in the boxed area in the bottom left corner. Scale bars: 25 μm. Data are shown as mean ± SD. *P < 0.05; **P < 0.01; ***P < 0.001; NS nonsignificant.

To explore whether TRPV1 can be a target to regulate macrophage polarization in OA, we first examined the expression of TRPV1 and M1 macrophage markers in the synovium of OA patients and rat OA model. Consistent with a previous study^7^, the number of CD14, CD80, and iNOS (M1 macrophage markers)-positive cells were significantly increased, and the proportion of TRPV1-positive cells was significantly higher in the human OA synovium than the human normal synovium (Fig. 1A, B). Macrophage accumulation in the 4- and 8-week-old rat OA model was then assessed. Compared to the sham group, macrophage infiltration in the synovium was increased in the OA groups, with a significantly higher percentage of CD14-positive cells (Fig. 1C, D). The proportion of M1 macrophage and TRPV1-positive cells were also significantly increased in the synovium of both 4- and 8-week-old OA models (Fig. 1C, D). In addition, the increased F4/80 (macrophage marker)-positive cells were observed in OA synovium (Supplementary Fig. 1A, C). However, the proportion of cells positive for CD163 and CD206 (M2 macrophage markers) showed no significant difference among each group (Supplementary Fig. 1A–D). These results suggest that M1 but not M2 macrophages accumulate in OA synovium, accompanied by significantly increased TRPV1 expression.

We next examined whether TRPV1 was expressed in macrophages, particularly M1 macrophages. The colocalization of CD80 and TRPV1 in rat OA synovium was identified, indicating that TRPV1 was expressed by the highly accumulated M1 macrophages (Fig. 1E). Besides, iNOS and TRPV1 were also highly co-localized. Of note, the percentage of TRPV1 localized to CD80- and iNOS-positive cells was >90% in the synovium of both sham and OA groups, but with no statistical difference (Fig. 1E), indicating that TRPV1 may be consistently expressed by M1 macrophages. Taken together, these findings suggest that TRPV1 is expressed by M1 macrophages and its expression is increased in the OA synovium, suggesting that TRPV1 could be a potential therapeutic target to regulate M1 macrophage polarization.

### TRPV1 alleviates OA by inhibiting M1 macrophage polarization

**Fig. 2:**
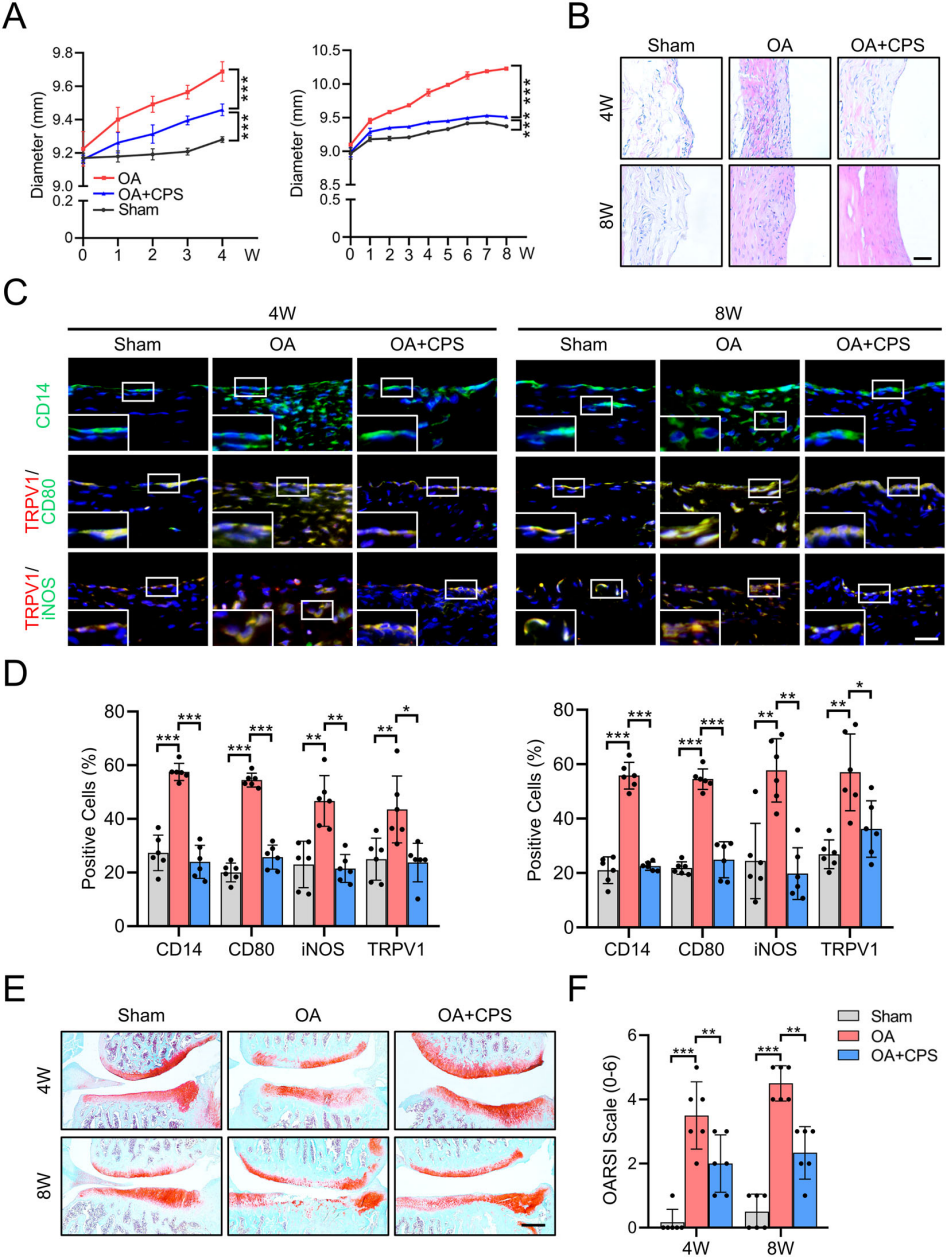
TRPV1 alleviates OA by inhibiting M1 macrophage polarization in vivo. **A** Time course of rat knee joint diameter in the sham, OA, and OA + CPS groups until 4 (left) and 8 (right) weeks after the radial transection of the medial meniscus. **B** H&E staining of the synovium in the 4- and 8-week-old sham, OA, and OA + CPS groups. Scale bars: 100 μm. **C** Immunofluorescence of CD14 and co-immunostaining of CD80 and iNOS with TRPV1 in the 4 and 8 weeks of sham, OA, and OA + CPS groups. Scale bars: 25 μm. Enlarged image is in the boxed area in the bottom left corner. **D** Quantification of CD14-, CD80-, iNOS-, and TRPV1-positive cells as a proportion of total cells in the synovium of 4- (left panel) and 8- (right panel) week sham, OA, and OA + CPS groups. **E** SO staining of the knee joint of 4- and 8-week-old sham, OA, and OA + CPS groups. **F** Quantitative analysis of the OARSI scale of 4- and 8-week-old sham, OA, and OA + CPS groups. Data (*n* = 6) are shown as mean ± SD. *P < 0.05; **P < 0.01; ***P < 0.001. W week, SO Safranin O/fast green.

To gain insight into whether the TRPV1 activation can inhibit M1 macrophage polarization and alleviate OA in vivo, we performed intra-articular injection of a specific TRPV1 agonist, CPS, in the rat OA model. After radial transection of the medial meniscus, the knee joint diameter of the OA groups increased faster than the sham group (Fig. 2A). However, after intra-articular injection of CPS, the diameter of rat knee joint increased much slower than the OA group, resulting in a significantly smaller knee diameter ultimately (Fig. 2A). Besides, there was no significant difference in the rat weight among every group during the entire experiment (Supplementary Fig. 2A).

The histological examination showed abundant cell infiltration and high levels of hyperplasia in the synovium of the 4- and 8-week-old OA groups, accompanied by significantly severe synovitis than the sham group (Fig. 2B and Supplementary Fig. 2B). After the CPS injection, the number of infiltrated cells and synovitis scores decreased significantly, although the synovium was still hyperplastic and hypertrophic (Fig. 2B and Supplementary Fig. 2B). Thus, intra-articular injection of a TRPV1 agonist decreased synovitis in OA rats. The percentage of macrophage in the rat synovium, particularly M1 macrophage, was significantly higher in the OA group compared to the sham group (Fig. 2C). Along with decreased synovitis scores, there was a substantial reduction in the number of CD14-, CD80-, iNOS- and TRPV1-positive cells in synovium following the CPS injection (Fig. 2C, D), indicating that TRPV1 attenuates M1 macrophage polarization.

We then investigated the role of inhibiting M1 macrophage polarization by TRPV1 activation in the development of OA. In the OA group, 4 weeks after surgery, larger area of the surface fibrillation, increased loss of cartilage extracellular matrix, and abnormal distribution of chondrocytes were observed compared to the sham group, although there was no discernible difference in the thickness of the articular cartilage (Fig. 2E and Supplementary Fig. 2C). Eight weeks after surgery, the articular cartilage was significantly thinner compared to the sham group (Fig. 2E). However, following intra-articular injection of CPS, improved score based on the OARSI scale (Fig. 2F), increased abundance of extracellular matrix, improved chondrocyte organization, a smoother cartilage surface, and less surface fibrillation were observed when compared to the OA group (Fig. 2E). We then evaluated the osteophyte formation by micro-CT analysis followed by three-dimensional (3D) modeling. Compared to the sham group, 4- and 8-week-old OA rats developed larger periarticular osteophytes (Supplementary Fig. 2D). However, the osteophytes were smaller in the CPS injection group than in the OA group. These findings suggest that TRPV1 activation induced by the intra-articular injection of a TRPV1 agonist can inhibit M1 macrophage polarization and thus protect from the development of OA.

### TRPV1 inhibits M1 macrophage polarization, proliferation, and migration in vitro

**Fig. 3:**
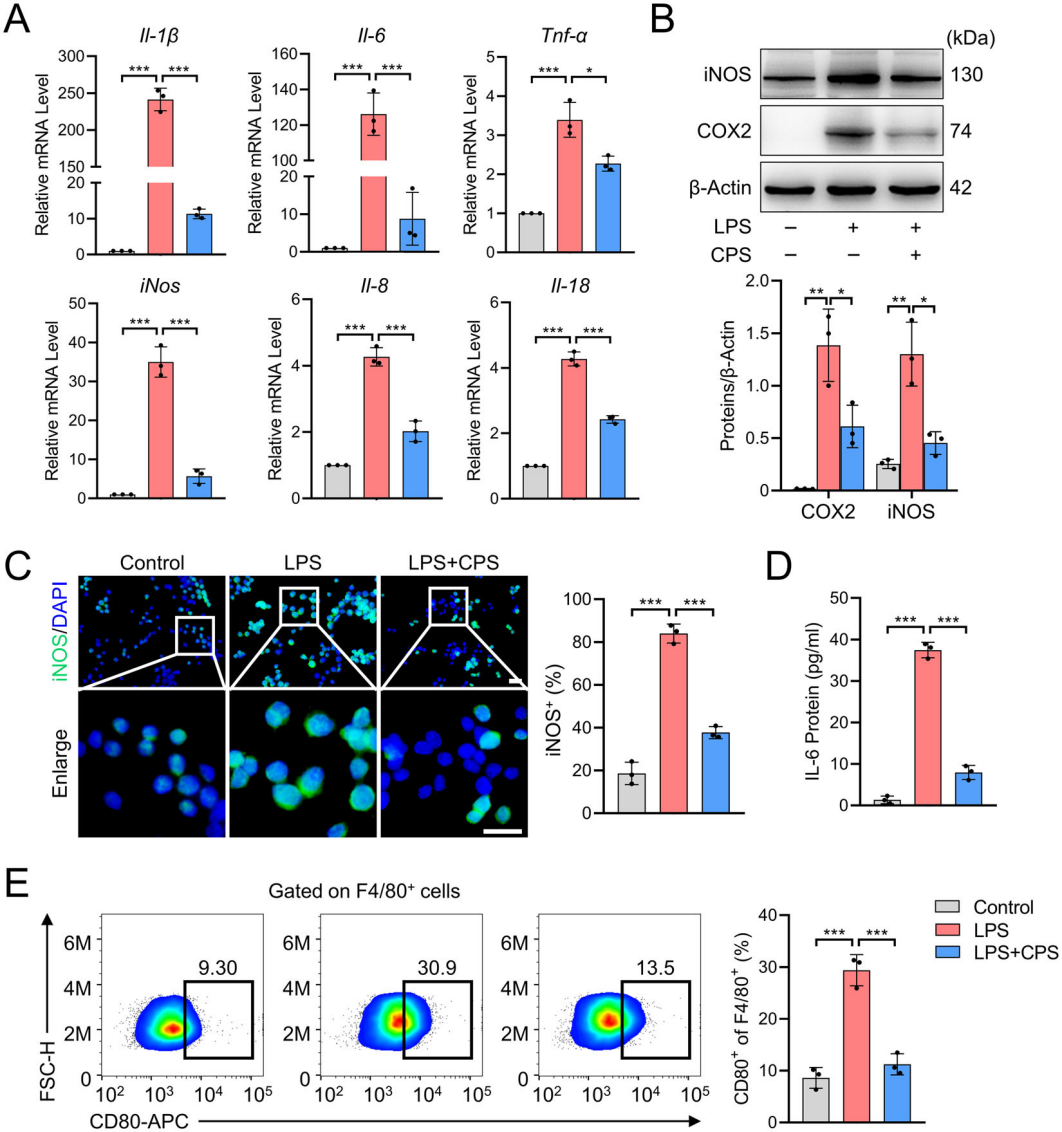
TRPV1 inhibits M1 macrophage polarization in RAW264.7 cells. **A** qPCR analysis of mRNA levels of M1 macrophage markers in the cells treated with LPS with or without CPS for 24 h. **B** Western blot and quantitative analysis of iNOS and COX2 proteins in the cells treated with LPS with or without CPS for 48 h. **C** Immunofluorescence and quantitative analysis of iNOS in RAW264.7 cells treated with LPS with or without CPS for 48 h. **D** ELISA analysis of IL-6 levels in the supernatant of the culture medium of RAW264.7 cells treated with LPS with or without CPS for 48 h. **E** Flow cytometry for detection and quantitative analysis of F4/80^+^CD80^+^ cells after the treatment of LPS with or without CPS for 48 h. Data (*n* = 3) are shown as mean ± SD. *P < 0.05; **P < 0.01; ***P < 0.001. Scale bars: 25 μm.

To explore the mechanism through which TRPV1 inhibits M1 macrophage polarization, we performed the subsequent experiments using LPS-treated RAW264.7 cells, a well-established M1 macrophage cell line^26,27^. First, we confirmed that the inflammatory factors, *Il-1β*, *Il-6*, *Tnf-α*, *iNos*, *Il-8*, and *Il-18*, were significantly increased in response to LPS stimulation at the mRNA level (Fig. 3A). iNOS and COX2 protein levels were also increased in LPS-treated cells compared to the vehicle control (Fig. 3B). Although activation of TRPV1 by CPS in LPS-treated RAW264.7 cells resulted in a significant reduction of inflammatory factors at mRNA and protein levels (Fig. 3A, B), it did not affect the expression of M2 macrophage markers (Supplementary Fig. 3A). Thus, we focused on the role of TRPV1 in M1 macrophage polarization. Diminished expression of iNOS in the cytoplasm of CPS-treated M1 macrophages was observed by immunofluorescence staining (Fig. 3C). IL-6, a soluble cytokine with a pleiotropic effect on inflammation and immune response, induces chondrocyte catabolism mainly via Stat3 signaling pathway^28,29^. Thus, we investigated whether TRPV1 activation can prevent the secretion of IL-6 from M1 macrophages. Using ELISA, we observed a massive secretion of IL-6 in the supernatant of M1 macrophage, which was significantly decreased by TRPV1 activation (Fig. 3D). Furthermore, we assessed the expression levels of M1 macrophage membrane markers by flow cytometry and demonstrated that the proportion of F4/80^+^CD80^+^ cells decreased remarkably after TRPV1 activation (Fig. 3E). These data indicate that TRPV1 can prevent the polarization of RAW264.7 cells towards the M1 macrophage phenotype promoted by LPS in vitro.

We further examined the role of TRPV1 in the proliferation and migration of M1 macrophage. After the treatment of CPS, the proliferation of LPS-induced M1 macrophage was significantly impeded (Supplementary Fig. 3B). EdU staining also demonstrated that the proliferation ability of M1 macrophage was significantly inhibited by TRPV1 activation (Supplementary Fig. 3C). Then, we investigated the efficiency of TRPV1 activation on the migration of macrophage. Under the treatment of LPS, the migration capacity of RAW264.7 cells was significantly enhanced, whereas the migration ability was remarkably suppressed by CPS treatment (Supplementary Fig. 3D–F). Besides, CPS inhibited the mRNA expression of (C–X–C motif) ligand 10 (*Cxcl10*) and monocyte chemoattractant protein-1 (*Mcp1*) (Supplementary Fig. 3G), two well-established chemokines of macrophage chemotaxis^30^. Together, our data indicate that TRPV1 activation by CPS plays an inhibitory role in the polarization, proliferation, and migration of LPS-induced M1 macrophage.

### TRPV1 promotes the nuclear translocation of Nrf2 and inhibits M1 macrophage polarization

**Fig. 4:**
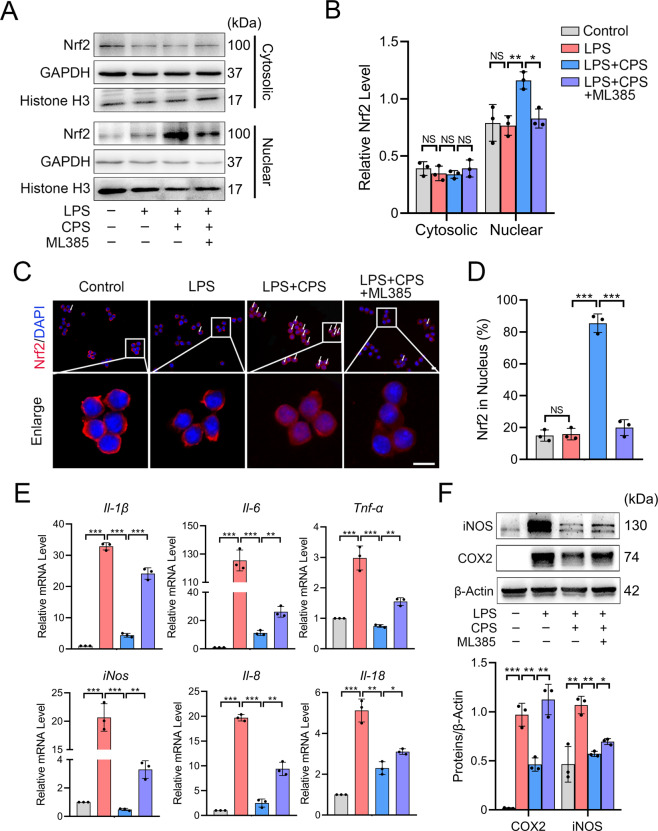
TRPV1 promotes the nuclear translocation of Nrf2 and inhibits M1 macrophage polarization in RAW264.7 cells. **A** Western blot and **B** quantitative analysis of cytosolic and nuclear Nrf2 protein levels in cells treated with LPS and LPS + CPS with or without ML385 for 3 h. **C** Immunofluorescence image of Nrf2 and **D** its quantitative analysis in RAW264.7 cells treated with LPS and LPS + CPS with or without ML385 for 3 h. White arrow: cells with Nrf2 nuclear translocation. **E** qPCR analysis of mRNA levels of M1 macrophage markers in RAW264.7 cells treated with LPS and LPS + CPS with or without ML385 for 24 h. **F** Western blot and quantitative analysis of iNOS and COX2 protein expression levels in RAW264.7 cells treated with LPS and LPS + CPS with or without ML385 for 48 h. Data (*n* = 3) are shown as mean ± SD. *P < 0.05; **P < 0.01; ***P < 0.001. Scale bars: 10 μm. NS nonsignificant.

The above results drove us to explore how TRPV1 inhibits M1 macrophage polarization. We examined whether TRPV1 could activate Nrf2, the master regulator of multiple cytoprotective responses that plays an anti-inflammatory role by repressing the transcription of proinflammatory genes and inhibiting the infiltration of immune cells^31^. Under normal conditions, Nrf2 is kept in the cytoplasm by Kelch-like ECH-associated protein-1 (Keap1), which facilitates its degradation; however, once activated, Nrf2 dissociates from Nrf2/Keap1 complex and translocates into the nucleus^32^. Therefore, we examined the nuclear level of Nrf2 after TRPV1 activation. Western blot analyses showed that the TRPV1 activation by CPS treatment significantly increased nuclear Nrf2, but not cytosolic Nrf2 (Fig. 4A, B). The rate of the nuclear localization of Nrf2 was significantly increased by TRPV1 activation (Fig. 4C). Over 80% of the cells analyzed displayed Nrf2 nuclear localization in the CPS treatment group, while the percentage in the control and LPS-treated groups was <20% (Fig. 4D), substantiating our conclusion regarding the effect of TRPV1 on Nrf2 activation. However, blocking the function of Nrf2 with its specific inhibitor, ML385, resulted in a significant decrease of density and positive rate of Nrf2 in the nucleus under the TRPV1 activation (Fig. 4A–D). These results indicate that Nrf2 mediates the downstream of the TRPV1 signal.

Next, we assessed whether Nrf2 is necessary for TRPV1-mediated inhibition of M1 macrophage polarization. mRNA expression of the M1 macrophage markers upregulated by LPS stimulation was significantly reduced by TRPV1 activation due to the CPS treatment, and ML385 partially blocked the effect of the CPS (Fig. 4E). Similar results were obtained in protein levels (Fig. 4F). Taken together, these results suggest that TRPV1 activation inhibits M1 macrophage polarization by activating the Nrf2 signaling pathway.

### TRPV1-mediated Ca^2+^ influx facilitates Nrf2 nuclear translocation

**Fig. 5:**
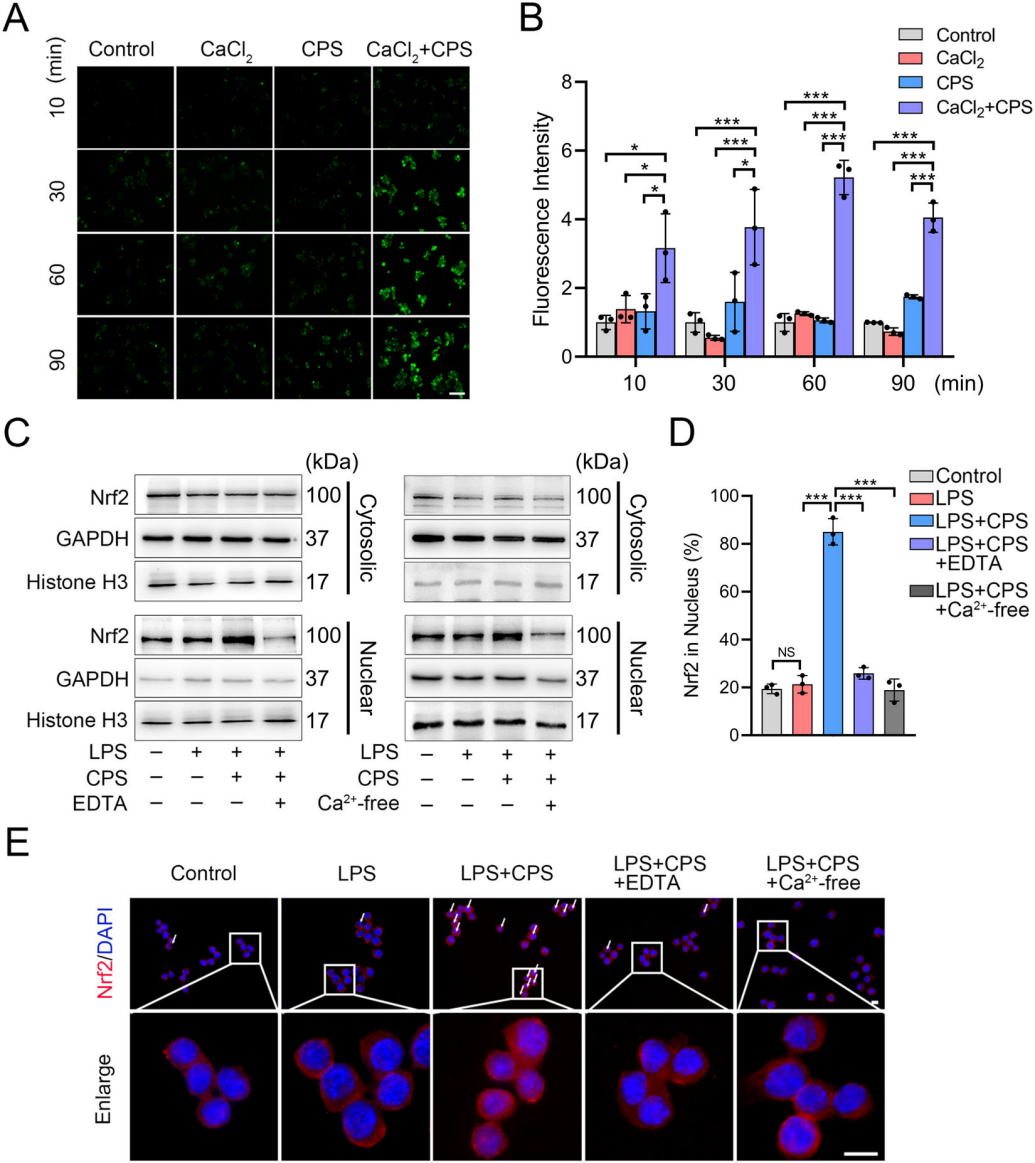
TRPV1-mediated Ca^2+^ influx promotes Nrf2 nuclear translocation in RAW264.7 cells. **A** Fluorescence images and **B** their quantitative analysis of intensity in the Fluo-4 AM-loaded RAW264.7 cells treated with CaCl_2_, CPS, or CaCl_2_ + CPS under the Ca^2+^-free medium for 10, 30, 60, and 90 min. Scale bars: 50 μm. **C** Western blot analysis of cytosolic and nuclear Nrf2 protein levels in the cells treated with LPS, LPS + CPS, and LPS + CPS with EDTA (left) or Ca^2+^-free medium (right) for 3 h. **D** Quantitative analysis and **E** representative images of Nrf2 immunofluorescence in the cells treated with LPS, LPS + CPS, and LPS + CPS with EDTA or Ca^2+^-free medium for 3 h. Scale bars: 10 μm. White arrow: cells with Nrf2 nuclear translocation. Data (*n* = 3) are shown as mean ± SD. *P < 0.05; ***P < 0.001; NS nonsignificant.

As a calcium-permeable channel, TRPV1 provides a path for the entry of calcium ions (Ca^2+^), an important second messenger in numerous cellular signaling pathways^10,33^. We reasoned that the TRPV1-mediated Ca^2+^ influx may regulate the Nrf2 nuclear translocation. Using a cell-permeable Ca^2+^ probe, Fluo-4 AM^34^, we examined the intracellular Ca^2+^ concentration with or without CPS and CaCl_2_ under the Ca^2+^-free condition. A significantly rapid increase in fluorescence intensity was observed after 10 min in the CPS + CaCl_2_ group, but not in the control, CaCl_2_-only, or CPS-only group, indicating that TRPV1-evoked Ca^2+^ influx (Fig. 5A, B). Of note, there was no statistically significant difference of fluorescence intensity among the control, CaCl_2_-only, and CPS-only groups (Fig. 5B), suggesting that other forms of Ca^2+^ influx that could interfere with the experimental results were absent.

We then investigated whether Ca^2+^ played a role in Nrf2 nuclear translocation and the M1 macrophage polarization. In the condition of TRPV1 activation, we used EDTA to chelate Ca^2+^ and observed a significant decrease in Nrf2 nuclear localization (Fig. 5C and Supplementary Fig. 4A). This result was also achieved by using Ca^2+^-free medium to fully remove Ca^2+^ (Fig. 5C and Supplementary Fig. 4B). Using immunofluorescence, we confirmed that both EDTA and Ca^2+^-free medium inhibited Nrf2 nuclear translocation (Fig. 5E). It is worth noting that the ratio of Nrf2-positive cells in the nucleus in the EDTA-treated and Ca^2+^-free group were similar to the control group, suggesting that TRPV1-mediated Nrf2 nuclear translocation may occur mainly through the Ca^2+^ influx via TRPV1 (Fig. 5D). Despite the TRPV1 activation, the expression of M1 macrophage markers at both the mRNA and protein levels was significantly increased after EDTA treatment (Supplementary Fig. 5A, B). These findings suggest that Ca^2+^ influx resulting from TRPV1 activation plays an indispensable role in the activation of Nrf2, which promotes the inhibition of M1 macrophage polarization.

### CaMKII mediates the Nrf2 nuclear translocation by TRPV1

**Fig. 6:**
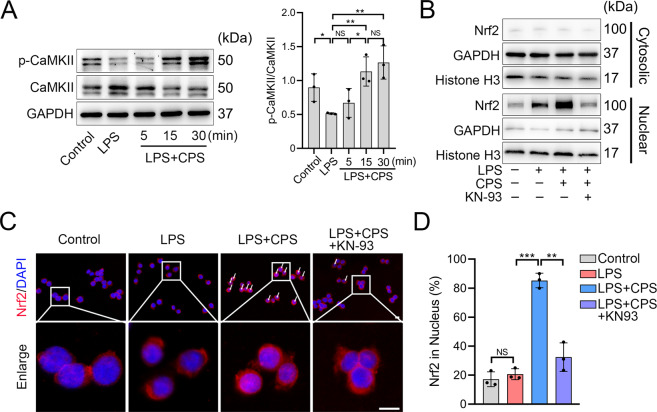
CaMKII mediates the TRPV1-promoted Nrf2 nuclear translocation in RAW264.7 cells. **A** Western blot and quantitative analyses of CaMKII and p-CaMKII protein levels in the cells treated with LPS with or without CPS for 5, 15, and 30 min. **B** Western blot analysis of cytosolic and nuclear Nrf2 protein levels in the cells treated with LPS and LPS + CPS with or without KN-93 for 3 h. **C** Immunofluorescence images and **D** quantitative analysis of Nrf2 in the cells treated with LPS and LPS + CPS with or without KN-93 for 3 h. Scale bars: 10 μm. Data (*n* = 3) are shown as mean ± SD. *P < 0.05; **P < 0.01; ***P < 0.001; NS nonsignificant.

Given the above results, we then examined a mediator that connects TRPV1-evoked Ca^2+^ influx and the Nrf2 nuclear localization. Given that CaMKII is a major Ca^2+^ signal transducer involved in a variety of signaling pathways^35,36^, we reasoned that CaMKII might be the mediator between Ca^2+^ signaling and Nrf2. We harvested proteins of RAW264.7 cells after 5-, 15-, and 30-min LPS induction with or without CPS treatment. Increased phosphorylation of CaMKII was observed after 15 and 30 min of the CPS treatment, along with a significant increase of p-CaMKII/CaMKII ratio, but not in the vehicle control- and LPS-treated groups (Fig. 6A), suggesting that CaMKII was activated following CPS treatment. KN-93^37^, a competitive inhibitor of p-CaMKII, significantly reduced the nuclear density of Nrf2 under the TRPV1 activation (Fig. 6B and Supplementary Fig. 6). Besides, the Nrf2 nuclear translocation was markedly decreased after the KN-93 treatment (Fig. 6C, D). Taken together, these findings indicate that CaMKII mediates the effect of TRPV1 activation on Nrf2 nuclear translocation.

## Discussion

In this study, we explored TRPV1 as a potential therapeutic target for inhibiting M1 macrophage polarization, proliferation, and migration in the pathogenesis of OA. We found that TRPV1 expression was upregulated in both human and rat OA synovium and was highly expressed by M1 macrophage. TRPV1 activation significantly decreased M1 macrophage polarization, reduced synovitis, and attenuated cartilage deterioration and osteophyte formation in the rat OA model. Furthermore, we unveiled that TRPV1 inhibited M1 macrophage polarization by Ca^2+^/CaMKII/Nrf2 signaling pathway.

Recent studies have shown that TRPV1 plays a key role in the management of pain due to its long-term desensitization effect on nociceptive nerve terminals^12,13^. A recent phase IIb clinical trial demonstrated that a single intra-articular injection of a TRPV1 agonist, CNTX-4975 (a type of CPS), provided a significant and clinically meaningful reduction of pain scores in patients with chronic OA-associated knee pain^15^. Based on this, topical application of CPS was conditionally recommended for OA in the 2019 American College of Rheumatology/Arthritis Foundation Guideline^38^. Beyond regulating pain responses, we showed that TRPV1 was involved in the regulation of macrophage polarization as it was highly localized to M1 macrophage, and the increase of the infiltrated M1 macrophage was highly correlated with synovitis and subsequent severity of OA^19^. However, the regulatory role of TRPV1, particularly in the context of M1 macrophage polarization, in OA remains unknown. Consistent with the previous studies^7,19^, we found markedly increased infiltration of M1 macrophages in human and rat OA synovium, accompanied by simultaneously upregulated TRPV1 expression. Approximately 90% of M1 macrophages expressed TRPV1 in both normal and OA synovium, providing a potential therapeutic target regulating M1 macrophage polarization precisely. Further, the rat OA model showed that TRPV1 activation attenuated knee joint swelling, improved synovitis scores, and reduced M1 macrophages levels, resulting in decreased cartilage degeneration and osteophyte formation. The results demonstrated that targeting TRPV1 is a potential therapeutic approach to prevent M1 macrophage polarization to reduce synovitis and slow OA progression. Further evaluation of the effect of TRPV1 on M1 macrophage polarization in the synovium of OA patients enrolled in clinical trials will contribute to a more comprehensive understanding of the therapeutic effect of targeting TRPV1 on synovitis.

Of note, TRPV1 is also found expressed in chondrocytes, and mediates the attenuation of cytokine-induced ADAMTS9 expression by mechanical strain, suggesting the decreased cartilage catabolism by TRPV1^39^. Thus, multiple pathological joint compartments and diverse processes involved in OA pathogenesis may respond to a TRPV1-targeting therapy.

Nrf2 is a redox-regulated transcription factor essential for the counteraction of oxidative stress and chronic inflammation^40,41^. Increased expression and enhanced activity of Nrf2 have been observed in the OA synovium and the damaged area of the OA cartilage, suggesting that there is a self-adaptive mechanism to promote cell survival in the OA microenvironment with increased inflammation and oxidative stress^42,43^. The current study identifies a protective role for Nrf2 in OA synovitis mediated via the inhibition of M1 macrophage polarization. TRPV1-evoked Ca^2+^ influx enhanced the phosphorylation of CaMKII and then activated Nrf2, leading to the downregulation of M1 macrophage markers. However, several recent studies have shown that ultraviolet irradiation induced the TRPV1-evoked Ca^2+^ influx and promoted Nrf2 degradation and cell apoptosis in human dermal fibroblasts^23,44^. The divergent effects could be explained by the dual role of Ca^2+^ in living organisms. Ca^2+^ signals are necessary for cell survival due to their management of a host of vital cell functions; however, Ca^2+^ influx caused by cytotoxic agents or receptor overstimulation can result in cytotoxicity and trigger either necrotic or apoptotic cell death^45^. Thus, we reason that the divergence in these studies following TRPV1 activation could be attributed to the dissimilar efficacy of CPS and ultraviolet irradiation on TRPV1 activity.

In conclusion, we found that TRPV1 is a novel therapeutic target for inhibiting M1 macrophage polarization and decreasing synovitis to attenuate OA progression, unveiling a new mechanism for the topical administration of CPS for the treatment of OA. Mechanically, CPS-mediated TRPV1 activation reduced M1 macrophage marker expression via the Ca^2+^/CaMKII/Nrf2 signaling pathway. Future studies are warranted to investigate the role of TRPV1 activation in chondrocytes, synovial fibroblasts, and other cells in the joint in order to generate a more comprehensive understanding of the pharmacological effects of intra-articular injection of CPS.

## Supplementary information

Supplementary table 1

Supplementary figure legends

Supplemetary figure 1

Supplemetary figure 2

Supplemetary figure 3

Supplemetary figure 4

Supplemetary figure 5

Supplemetary figure 6
