# Supplementary material for: TRPV1 alleviates osteoarthritis by inhibiting M1 macrophage polarization via Ca2+/CaMKII/Nrf2 signaling pathway
Source: Cell Death Dis. 2021 May 18;12(6):504. doi: 10.1038/s41419-021-03792-8 (PMC8131608; doi:10.1038/s41419-021-03792-8)
Supplement: Supplementary file 1 — Supplementary table 1 [file 41419_2021_3792_MOESM1_ESM.docx]

**TRPV1 Alleviates Osteoarthritis by Inhibiting M1 Macrophage Polarization *via* Ca^2+^/CaMKII/Nrf2 Signaling Pathway**

Lv et al.

**Supplementary Table 1**

Primer sequences for genes tested with RAW264.7 cells.

| Gene | Forward | Reverse |
| --- | --- | --- |
| *Il-1β*  *Il-6*  *Tnf-α*  *iNos*  *Il-8*  *Il-18*  *Cxcl10*  *Mcp1* | 5′-TGCCACCTTTTGACAGTGATG-3′  5′-TAGTCCTTCCTACCCCAATTTCC-3′  5′-CCACCATCAAGGACTCAA-3′  5′-GCGCTCTAGTGAAGCAAAGC-3′  5′-AGTGAAATCCGATGTGGCCT-3′  5′-GAAAGCCGCCTCAAACCTTC-3′  5′-CCACGTGTTGAGATCATTGCC-3′  5′-GGGCCTGCTGTTCACAGTT-3′ | 5′-TGCCACCTTTTGACAGTGATG-3′  5′-TTGGTCCTTAGCCACTCCTTC-3′  5′-CAGGGAAGAATCTGGAAAGG-3′  5′-AGTGAAATCCGATGTGGCCT-3′  5′-CAGAAGCTTCATTGCCGGTG-3′  5′-GGTGGATCCATTTCCACTTTGA-3′  5′-GAGGCTCTCTGCTGTCCATC-3′  5′-CCAGCCTACTCATTGGGAT-3′ |
