# Supplementary material for: TRPV1 alleviates osteoarthritis by inhibiting M1 macrophage polarization via Ca2+/CaMKII/Nrf2 signaling pathway
Source: Cell Death Dis. 2021 May 18;12(6):504. doi: 10.1038/s41419-021-03792-8 (PMC8131608; doi:10.1038/s41419-021-03792-8)
Supplement: Supplementary file 2 — Supplementary figure legends [file 41419_2021_3792_MOESM2_ESM.docx]

**TRPV1 Alleviates Osteoarthritis by Inhibiting M1 Macrophage Polarization *via* Ca^2+^/CaMKII/Nrf2 Signaling Pathway**

Lv et al.

**Supplementary Figure Legends**

**Supplementary Figure 1. F4/80, CD163 and CD206 expression in the synovium of OA patients and rat OA model.** (A) Immunofluorescence images for F4/80, CD163 and CD206 in human normal and OA synovium. (B) Quantification of F4/80, CD163 and CD206-positive cells as a proportion of total cells in normal (n = 3) and OA (n = 6) human synovium. (C) Immunofluorescence images for F4/80, CD163 and CD206 in the synovium of sham and rats at 4 and 8 weeks after the radial transection of the medial meniscus. (D) Quantification of F4/80, CD163 and CD206-positive cells as a proportion of total cells in the synovium of sham and 4- and 8-week OA rats (n = 6). Enlarged image is in the boxed area in the bottom left corner. Scale bars: 25 μm. Data are shown as mean ± SD. *** P < 0.001; NS, no significant.

**Supplementary Figure 2. Effect of the intra-articular injection of capsaicin (CPS) for the surgically induced rat OA model.** (A) Time course of the weight in the sham, OA, and OA+CPS groups until 4 and 8 weeks after the radial transection of the medial meniscus. (B) Quantification of synovitis score in the 4- and 8-week sham, OA, and OA+CPS groups. (C) Histological changes of the knee joints at 4 and 8 weeks (W) after surgery in the sham, OA, and OA+CPS groups (H&E staining). Scale bars: 400 μm. (D) Frontal and lateral views of micro-CT scan and 3D reconstruction of the knee joint of the sham, OA, and OA+CPS groups 4 and 8 weeks after surgery. Scale bar: 2 mm. Data (n = 6) are shown as mean ± SD. ** P < 0.01; *** P < 0.001.

**Supplementary Figure 3. Effects of capsaicin (CPS) on the expression of M2 macrophage markers, the proliferation and migration in lipopolysaccharide (LPS)-induced RAW264.7 cells.** (A) qPCR analysis of mRNA levels of M2 macrophage markers, *Cd206*, *Arg-1* and *Il-10* in RAW264.7 cells treated with LPS with or without CPS for 24 h. (B) Alamar Blue and CCK8 analysis of cell proliferation after the treatment of LPS with or without CPS for 24 h. (C) EdU staining and quantitative analysis of EdU^+^ cells after the treatment of LPS with or without CPS for 24 h. Scale bar: 50 μm. (D) Wound scratch assay for the assessment of RAW264.7 cells migration capacity after treated with LPS with or without CPS for 0, 12, 24 and 48 h. Scale bar: 200 μm. (E) Transwell assay of RAW264.7 cells migration and quantitative analysis (F) of migrated cells with the treatment of LPS with or without CPS for 24 h. Scale bar: 100 μm. (G) qPCR analysis of mRNA levels of *Cxcl10* and *Mcp1* with the treatment of LPS with or without CPS for 24 h. Data (n = 3) are shown as mean ± SD. * P < 0.05; ** P < 0.01; *** P < 0.001; NS, no significant. OA, osteoarthritis; Arg-1, arginine-1; Il-10, interlukine-10; Cxcl10, (C-X-C motif) ligand 10; Mcp1, monocyte chemoattractant protein-1.

**Supplementary Figure 4. Ca^2+^ depletion decreased the TRPV1-activated Nrf2 nuclear translocation.** Quantitative analysis of cytosolic and nuclear Nrf2 protein levels in RAW264.7 cells treated with lipopolysaccharide (LPS), LPS+ capsaicin (CPS), and LPS+CPS with (**A**) ethylene diamine tetra-acetic acid (EDTA) or with (**B**) Ca^2+^-free medium for 3 h. Data (n = 3) are shown as mean ± SD. * P < 0.05; ** P < 0.01; *** P < 0.001, NS, no significant.

**Supplementary Figure 5. Ca^2+^ depletion attenuates the effect of TRPV1 on M1 macrophage polarization.** (**A**) qPCR analysis of mRNA levels of M1 macrophage markers in the cells treated with lipopolysaccharide (LPS), LPS+ capsaicin (CPS), and LPS+CPS with ethylene diamine tetra-acetic acid (EDTA) for 24 h. (B) Western blot and quantitative analyses of iNOS and COX2 proteins in the cells treated with LPS, LPS+CPS and LPS+CPS with EDTA for 48 h. Data (n = 3) are shown as mean ± SD. * P < 0.05; ** P < 0.01; *** P < 0.001.

**Supplementary Figure 6. KN-93, a p-CaMKII inhibitor significantly reduced the nuclear density of Nrf2 under TRPV1 activation.** Quantitative analysis of cytosolic and nuclear Nrf2 protein levels in RAW264.7 cells treated with lipopolysaccharide (LPS), LPS+ capsaicin (CPS) with or without KN-93 for 3 h. Data (n = 3) are shown as mean ± SD. * P < 0.05; ** P < 0.01; NS, no significant.
