# Supplementary figures and images for: TRPV1 alleviates osteoarthritis by inhibiting M1 macrophage polarization via Ca2+/CaMKII/Nrf2 signaling pathway
Source: Cell Death Dis. 2021 May 18;12(6):504. doi: 10.1038/s41419-021-03792-8 (PMC8131608; doi:10.1038/s41419-021-03792-8)

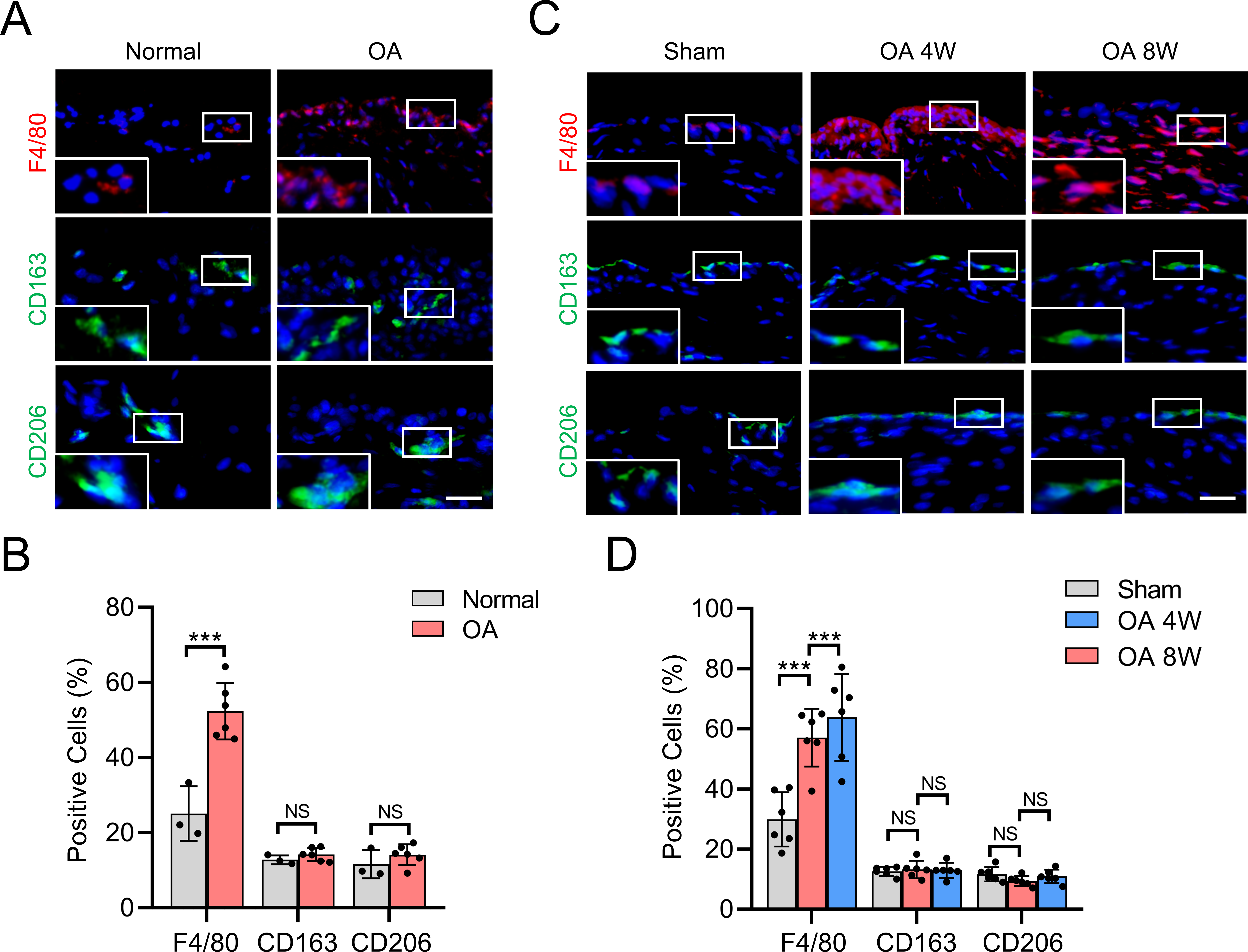

Supplement: Supplementary file 3 — Supplemetary figure 1 [file 41419_2021_3792_MOESM3_ESM.tif]

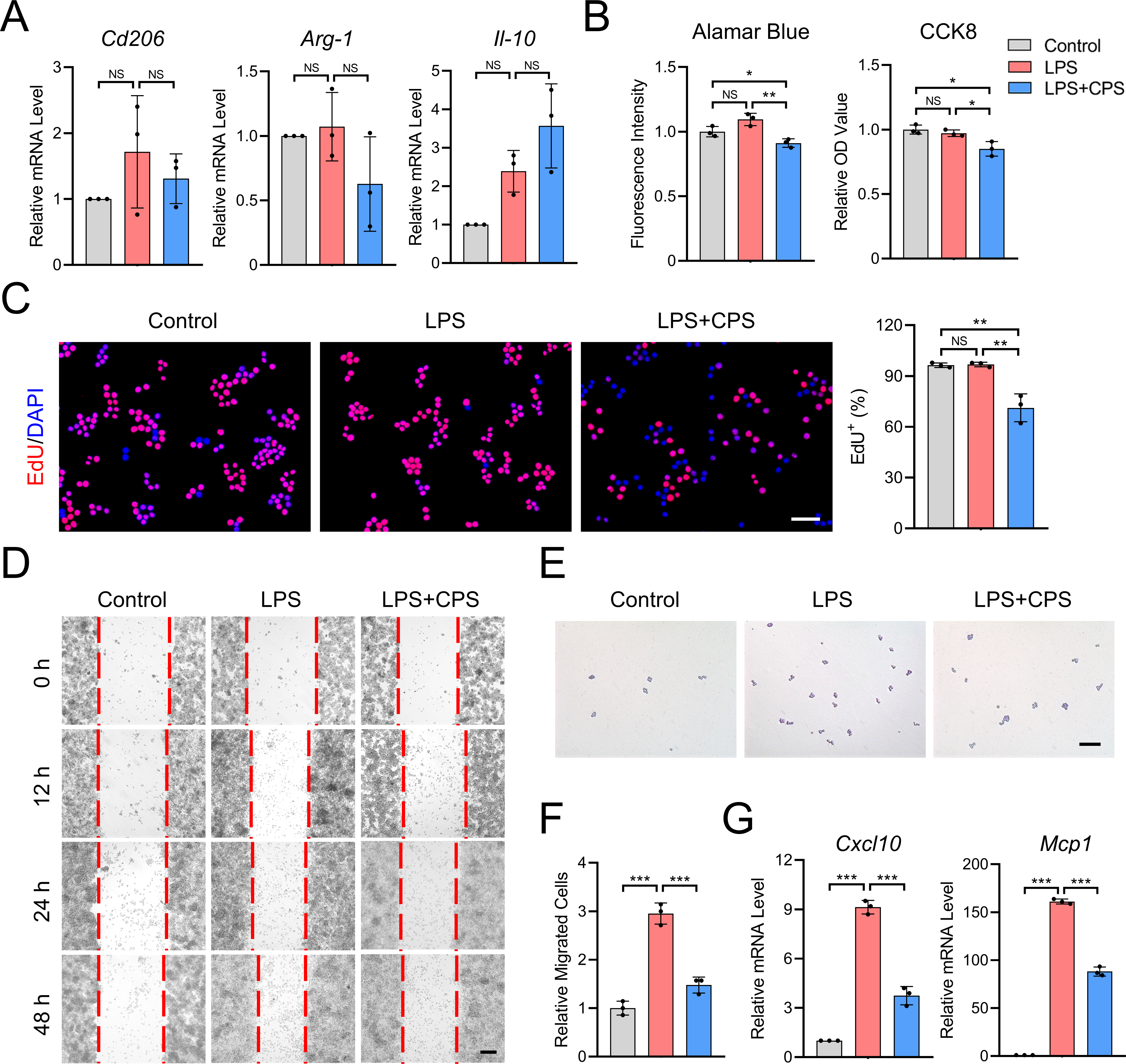

Supplement: Supplementary file 5 — Supplemetary figure 3 [file 41419_2021_3792_MOESM5_ESM.tif]

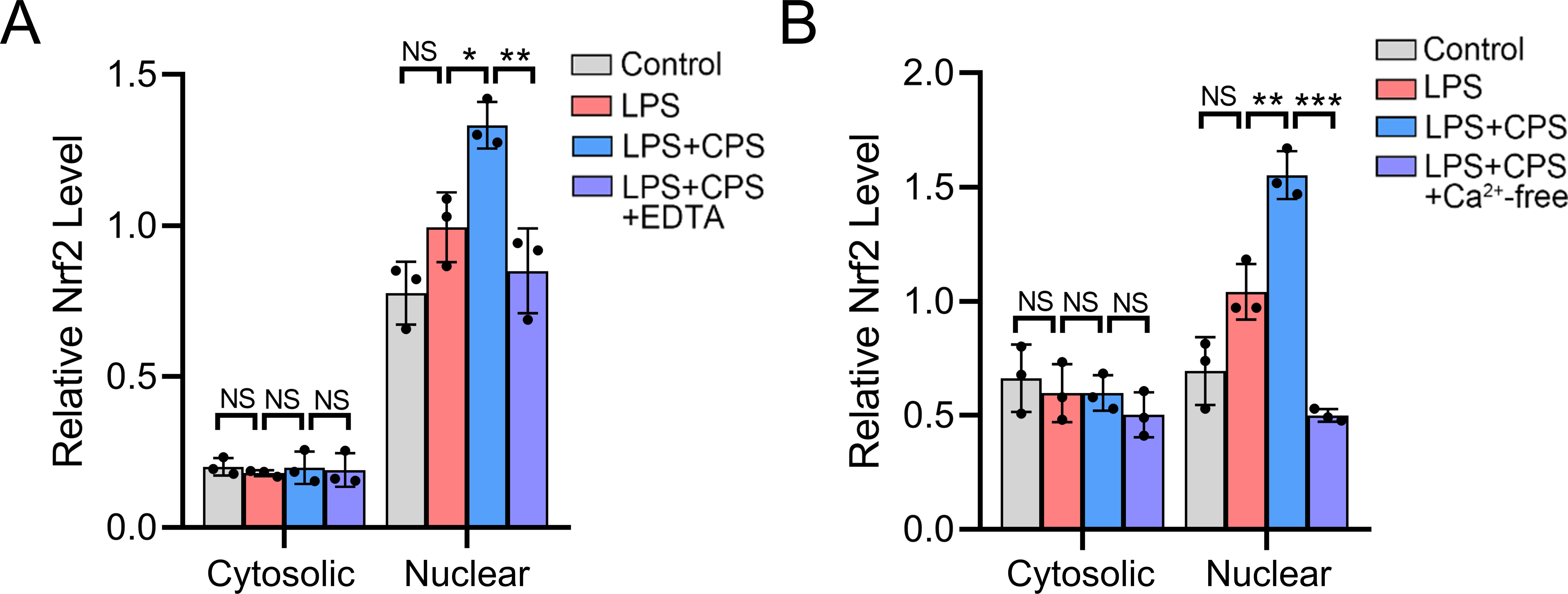

Supplement: Supplementary file 6 — Supplemetary figure 4 [file 41419_2021_3792_MOESM6_ESM.tif]

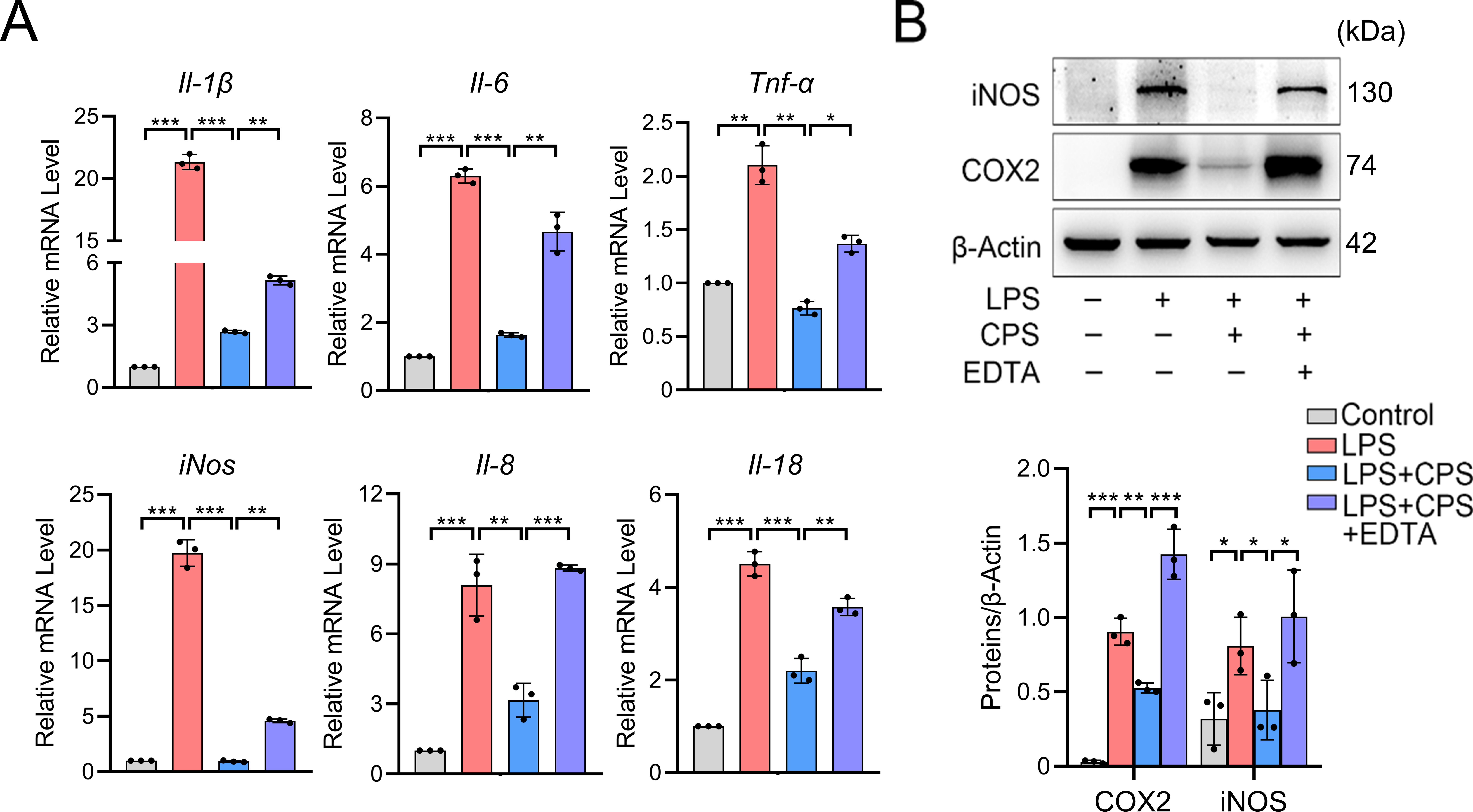

Supplement: Supplementary file 7 — Supplemetary figure 5 [file 41419_2021_3792_MOESM7_ESM.tif]

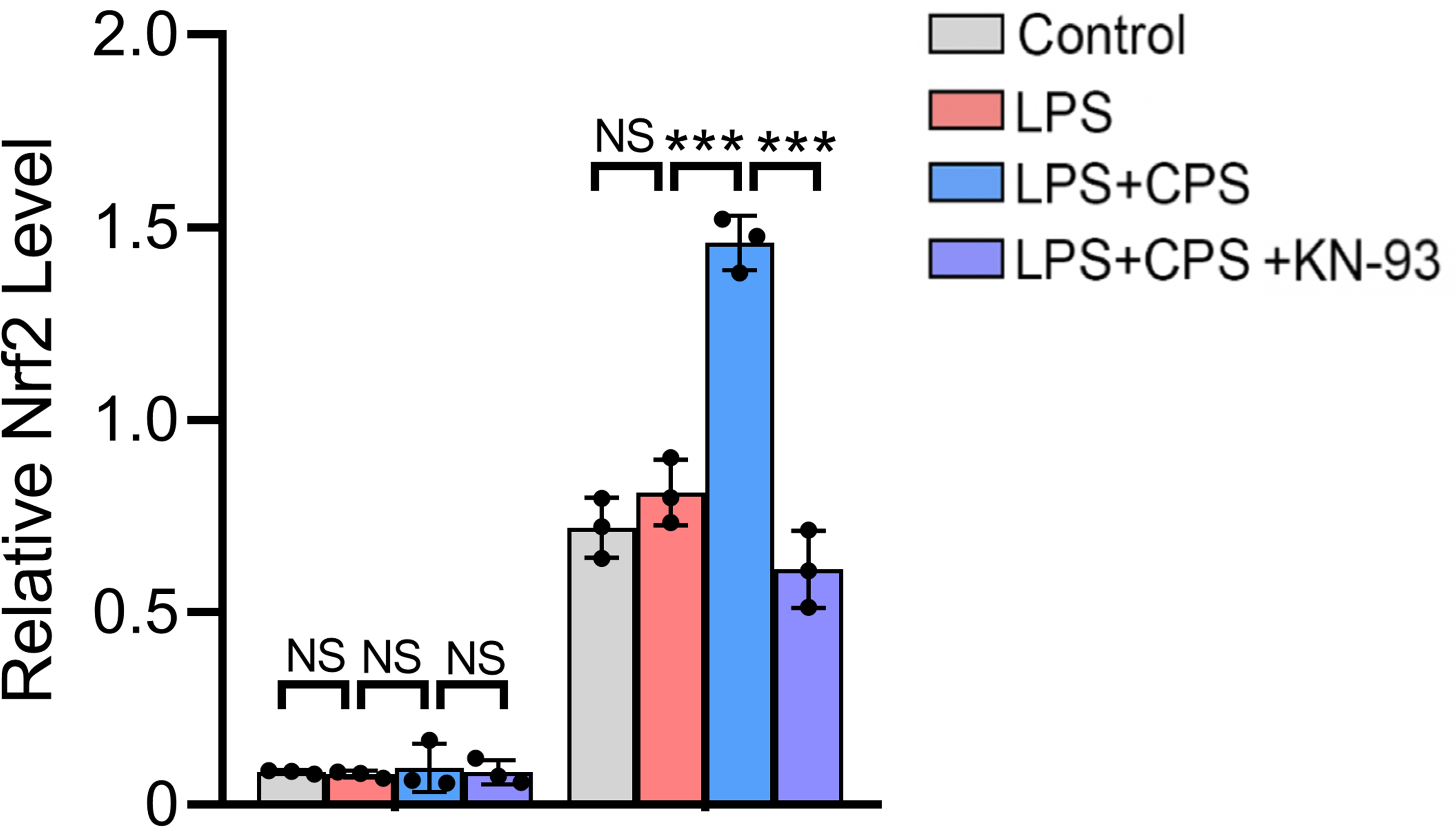

Supplement: Supplementary file 8 — Supplemetary figure 6 [file 41419_2021_3792_MOESM8_ESM.tif]
